# Reliability and validity of the Baecke physical activity questionnaire in adult women with hip disorders

**DOI:** 10.1186/1471-2474-8-61

**Published:** 2007-07-05

**Authors:** Rei Ono, Soichiro Hirata, Minoru Yamada, Takayuki Nishiyama, Masahiro Kurosaka, Yumi Tamura

**Affiliations:** 1 Faculty of Health Sciences, Kobe University School of Medicine, 7-10-2, Tomogaoka, Suma-Ku, Kobe, 654-0142, Japan; 2 Department of Orthopaedic Surgery, Kobe University Graduate School of Medicine, 7-5-2, Kusunoki-cho, Chuo-ku, Kobe, 650-0017, Japan; 3 Department of Epidemiology and Healthcare Research, Graduate School of Medicine and Public Health, Kyoto University, Yoshida-Konoe-cho, Sakyo-Ku, Kyoto 606-8501, Japan

## Abstract

**Background:**

Although physical activity maintenance is important for OA management, it is not clear whether people with OA are more inactive or not. One possible reason is no simple monitoring tool to assess physical activity at the clinic. The aim of this study was to determine the reliability and validity of the Baecke Physical Activity Questionnaire (BQ) in adult women with hip disorders.

**Methods:**

Sixty-four patients with unilateral or bilateral hip disorders were recruited from an outpatients clinic at a university hospital in Japan. BQ includes a total of 16 questions classified into three domains: work, sports, and non-sports leisure activity. For test-retest reliability, one BQ was administrated face-to-face, and a second was mailed to participants two weeks later. Test-retest reliability of BQ was assessed using intra-class correlation (ICC) and Bland and Altman method. To determine criterion validity, the correlation between BQ measurements and pedometer-measured step counts was assessed. Correlations between BQ measurements and step counts were assessed using Spearman rank correlation coefficient (rho).

**Results:**

Analyses were restricted to the 61 patients (53.3 ± 11.3 years old) who wore the pedometer continuously for 5 days or more. Twenty eight patients had unilateral hip osteoarthritis, 17 patients had unilateral total hip arthroplasty, and 16 patients had hip osteoarthritis and total hip arthroplasty. The mean step count was 6,309 ± 2,392 steps/day. In analysis for reliability, the value of ICC was 0.84 for work, 0.83 for sports, 0.78 for non-sports leisure activity, and 0.87 total. Bland and Altman analysis showed the step count and BQ total did not differ significantly from 0 with most falling between 0 ± 1.96 SD. In analysis for validity, there was a significant but low to moderate correlation between step counts and 3 BQ subscales (rho, 0.30–0.49) and a higher correlation between step counts and total score (rho, 0.49).

**Conclusion:**

BQ is a useful monitoring tool for assessing multiple domains of physical activity with acceptable reliability and validity in adult women with hip disorders.

## Background

Osteoarthritis (OA) is the most common form of arthritis and is a leading cause of mobility-related disability in older adults [1]. The current American College of Rheumatology guidelines for OA management stress the importance of non-pharmacologic methods of reducing pain and improving function including weight loss if overweight and aerobic exercise [2]. Despite these recommendations, there is a significantly higher prevalence of no physical activity among people with OA, as compared with the general population [3]. Similarly, people with arthritis have an overall lower physical activity profile than those without arthritis [4]. These findings may partly explain the aerobic capacity and reduced muscle strength and range of motion among people with OA [5].

It is well established that physical inactivity is a risk factor for cardiovascular disease, diabetes, osteoporosis, and some cancers [6]. Therefore, patients with OA may be at a higher risk of those diseases. Indeed, Singh G and Gebriel S [7] estimated that cardiovascular disease is prevalent among people with OA. The promotion of regular physical activity may, thus, not only improve joint function but also prevent inactivity-related disease in adults with OA.

Physical activity, however, is a complex and multidimensional exposure variable, making population-based measurement difficult [8]. Although there is no universally accepted gold standard for measuring physical activity, some direct and indirect measurement techniques exist, such as activity monitor using an accelerometer, heart rate monitor, double labeled water, and questionnaires. Each method has its advantage and disadvantage but administered questionnaires have become an invaluable tool for physical activity research because of their minimal expense and scoring flexibility. Information from questionnaires allows the greatest flexibility in providing behavioral descriptions of physical activity patterns and/or more quantitative summary estimates of energy expenditure due to physical activity [9].

Among many questionnaires, the Baecke Questionnaire (BQ) [10] is one of the most widely used tools for assessing older adults with chronic conditions [11-13]. BQ is a simple, short questionnaire that is easy to self-administer making it a very attractive assessment tool for routine use in a busy clinical setting. However although the original BQ was validated in young people, it was then modified for elderly people with and without chronic disease [14-16] and to our knowledge, its validity and reliability has not been investigated in older adults with hip disorders, including OA and post-total hip arthroplasty (THA). The aim of this study is to examine the reliability and validity of BQ in Japanese adult women with these hip disorders.

## Methods

### Subjects

Sixty four consecutive participants were recruited when visiting an outpatient's clinic at a university hospital. The inclusion criteria were adult women with OA in one or both hips who had or had not undergone past salvage surgery such as femoral osteotomy, and those who had undergone THA for pre-existing OA. All patients were community-dwelling and receiving no regular nursing care. Patients were limited to women because of their great predominance in the clinic. Radiographic evidence of joint space narrowing and associated bony changes confirmed the presence of OA. All surgeries were performed at least 6 months before study entry so that rapid functional changes, which usually occur in the six month postoperative period, could be minimized during the study. The exclusion criteria were symptoms in other weight-bearing joints, and/or chronic diseases which could possibly affect mobility. As all data were collected as part of medical care, no ethical approval was requested. All patients were given experimental information in compliance with Helsinki Declaration and only those who gave consent participated in the study.

### Measures

The Harris Hip Score (HHS) was used to evaluate overall function of the hip, due to its wide use as an outcome measurement after hip surgery. HHS has 4 domains: function, pain, deformity, and range of motion, allowing a total score ranging from 0 to 100 points (low-high), which are categorized as follows: 0 to 70 points, poor; 70 to 80 points, fair; 80 to 90 points, good; 90 to 100 points, excellent [17].

BQ includes a total of 16 questions classified into three domains: work, sports, and non-sports leisure activity. Each domain has several questions scored on a five-point Likert scale, ranging from never to always or very often. Scoring of the questionnaire in our study followed the original system; work was the mean score among eight occupational questions, sports was the mean score among four sports-related questions, and non-sports leisure was the mean score among four habitual physical activities during leisure time. Each domain could receive a score from one to five points, thus allowing a total score from three (minimum) to fifteen (maximum). For the two most frequently reported sports activities, specific questions regarding the number of months per year and hours per week of participation were addressed. The original BQ was translated into Japanese using the forward and backward translation procedure. Two professional translators independently translated the original scale once. Synthesis of the translations was conducted by all members of the study team. For test-retest reliability, the initial BQ was administered during face-to-face consultations, and a second was mailed to participants two week later, and they were asked to return it by mail within a few days. Three subscales "work", "sports", "non-sports leisure" were scored and totaled.

Participants were asked to wear a digital pedometer with a uniaxial acceleration sensor (Lifecorder, Suzuken Co., Nagoya, Japan) at the waist, during waking hours except during bathing or swimming and to do ordinary daily activities without reading the step count. A pedometer estimates the number of steps taken based on acceleration signals. Although the reproducibility and validity of the pedometer in counting walking steps has been established in healthy people [18], we confirmed its accuracy for step counting in our patients. Patients wore pedometers at the waist and walked about 100 steps at their usual pace along a corridor. Accuracy was calculated by count increase while walking divided by exact number of steps taken as measured using a hand-tally. The test confirmed the accuracy with a mean accuracy of 94.3% (SD, 5.9). Patients wore the pedometers for nine days, starting on the day of their visit, and then returned them by mail in a cushioned envelope. Physical activity data was transferred to a personal computer for calculations. Physical activity was evaluated as the average number of steps taken per day over 7 days (the first and last days of the measurement were excluded).

### Statistical analysis

Test-retest reliability of BQ was assessed using intra-class correlation (ICC) [19] and Bland and Altman method [20] as these assessments give complementary information [21,22]. ICC has a range of 0 (totally unreliable) to1 (completely reliable). As a general guideline, ICC values above 0.75 indicate good reliability and those below 0.75 indicate poor to moderate reliability [23]. Reliability was assessed separately for subscales of work, sport, and non-sports leisure, and also for total BQ. To determine criterion validity, correlations between BQ measurements and step counts were assessed using Spearman rank correlation coefficient (rho). All statistical analyses were performed using computer software (SPSS 15.0J, SPSS Japan Inc., Tokyo, Japan), and P values < 0.05 were considered statistically significant.

## Results

### Activity monitor-wearing

Of 64 patients, 46 were considered to have worn the pedometer continuously from morning until night for 7 days, 9 for 6 days, 6 for 5 days, 2 for 4 days, and 1 for 3 days during the measurement period. Analysis were restricted to the 61 patients data who wore the pedometer for 5 days or more,

### Subject characteristics

Table 1 shows the demographic and clinical characteristics of the patients. The sixty-one participants had a mean age of 53.3 years (standard deviation, 11.3) and a mean BMI (weight divided by square meters of height) of 22.0 (3.0) kg/m^2^. Twenty eight patients (46%) had unilateral hip osteoarthritis, 17 patients (28%) had unilateral total hip arthroplasty, and 16 patients (26%) had hip osteoarthritis and total hip arthroplasty. The mean step count was 6309 (2392) steps/day. The mean HHS score was 79.3 (12.7). Of the 61 patients, 16 (26%) were categorized as poor HHS, 15 (27%) were fair, 16 (26%) were good, and 14 (23%) were excellent. The mean subscales of initial BQ were 2.8 (SD 0.6) for work, 2.1 (SD 0.6) for sports, 2.6 (SD 0.6) for non-sports leisure, and 7.5 (SD 1.4) for total. From initial BQ, 23 patients (38%) reported engagement in sports. Eighteen of them (78%) were engaged in aqua exercise, such as swimming or aqua walking. The other sports were muscle strengthening exercises and using an exercise bike.

**Table 1:** Demographics and clinical characteristics of 61 patients

Variables			Mean (SD)	Number (%)
Age (years)			53.3 (11.3)	
boby mass index (kg/m2)			22.0 (3.0)	
step counts (steps/day)			6309 (2392)	
hip involvement	unilateral	OA		28 (46)
		THA		17 (28)
	bilateral	OA and THA		16 (26)
HHS category	poor			16 (26)
	fair			15 (25)
	good			16 (26)
	excellent			14 (23)

Harris Hip Score (HHS): Poor; < 70; Fair; < 80; Good, < 90; Excellent, ≧ 90

OA: Osteoarthritis

THA: Total Hip Arthroplasty

SD: Standard Deviation

### Reliability

**Figure 1:**
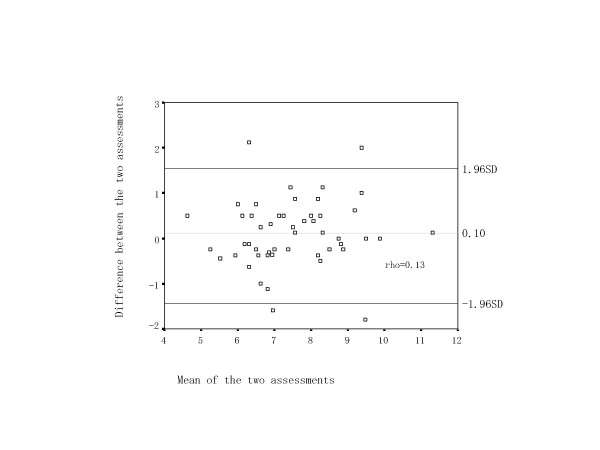
Baecke Questionnaire total score reliability. Broken line shows mean difference in BQ total score. Solid lines show ± 1.96 × standard deviation of BQ total score. Rho: Spearman's correlation between the mean and difference of the two assessments.

Of the 61 participants who completed the first BQ, 52 (85%) returned the second BQ. The nine patients who did not return it had no specific characteristics of age, HHS, or step counts. Table 2 shows means, SDs, and ranges of the first and second BQ measurements, and ICC (n = 52). The values of ICC for three subscales and the total score were all > 0.75, reaching a substantial level. In BQ total score, Bland and Altman analysis showed no significant difference between the two measurements with most falling between 0 ± 1.96 SD (Figure 1). Furthermore, no systematic trends of correlation were observed (rho = 0.13).

**Table 2:** Baecke Questionnaire subscales, total score, and ICC

Variables		measurement1	measurement2	ICC (95%CI)
			
		mean(SD)	mean(SD)	
		n = 61	n = 52	n = 52
BQ subscales	Work	2.8 (0.6)	2.8 (0.6)	0.84 (0.73 – 0.90)
	Sport	2.1 (0.6)	2.0 (0.7)	0.83 (0.72 – 0.90)
	Non-sports leisure	2.6 (0.4)	2.7 (0.5)	0.78 (0.65 – 0.87)
BQ total		7.6 (1.4)	7.6 (1.4)	0.87 (0.78 – 0.92)

Iintra-class correlation coefficient (ICC)

### Criterion validity

Table 3 shows the relationship of step count with BQ subscales and total score. There was significant but low to moderate correlation between step count and 3 BQ subscales (rho, 0.30–0.49) and a higher correlation between step counts and total score (rho, 0.49).

**Table 3:** Spearman's rank correlation coefficients between Baecke Questionnaire and pedometer

Variables		step counts
BQ subscales	work	0.42**
	sports	0.30**
	non-sports leisure	0.42**
BQ total		0.49**

*p < 0.05 **p < 0.01

## Discussion

This study first evaluated the reliability and validity of physical activity questionnaire in adult women with hip osteoarthritis, total hip arthroplasty, or both. BQ showed an acceptable reliability and validity and may be a useful tool for assessing physical activity in patients with hip disorders.

Three subscales and total score of BQ showed high correlation coefficients between test and re-test, which is consistent with the literature [10,24]. In the present study, ICC was additionally assessed because Pearson's correlation measures the strength of a relationship between two measurements, not the agreement between them [20]. The values of ICC were at substantial levels. Philippaerts and colleague [25] reported that the values of ICC were 0.86 to 95 for BQ 3 subscales and total in 90 Flemish male. Jacob and colleague [24] also reported in 151 patients with low back pain that the values of ICC were above 0.89 for BQ 3 subscales and total. The values of ICC in this study were slightly lower than those of previous studies. This may be because the first and second BQ were completed in a different way (the first was face-to face and second was mailed). However, ICC values above 0.75 are considered reliable [23] and acceptable. Furthermore, Bland and Altman analysis showed no systematic bias or significant random error on total BQ between test and re-test. These results suggest that BQ is reliable for assessing physical activity in adult women with hip disorders.

We used step count assessment by pedometer as a reference tool. Walking is the most frequently reported leisure-time physical activity in patients with arthritis [26]. Additionally, no previous studies have validated BQ using a pedometer, although the pedometer has been previously shown to be valid with physical activity questionnaires [27,28]. The numbers of steps taken also showed a close relationship to measures of questionnaire responses in a large, free-living population [27]. Questionnaire scores were highly correlated with pedometer scores in older adults [28]. The pedometer model we used proved to be one of the most accurate brands [29] and, in the present study, had a good mean accuracy of 94.3% in step counting regardless of hip disorders.

The magnitude of BQ correlations varies substantially among different study samples and instruments measuring physical activity in the literature. For example, a total score of BQ was highly correlated with a measure using double labeled water (Pearson's r = .69), the gold standard in the assessment of physical activity, in men aged 40 years [30]. However, as in our study, lower correlation coefficients of BQ measures were reported for other instruments: .38 to .46 for peak oxygen consumption, .06 to.24 for accelerometer readings, .24 to .42 for a 48-hour diary of physical activity in women aged 21–59 years [31], and .42 or .44 for a 3-day diary in women aged 20–70 years [32]. In the present study, although BQ measures were low to moderately correlated with step counts, the correlation coefficients found in this study were higher than those reported in studies above mentioned except double labeled water. The result suggested that BQ is an acceptable questionnaire in hip disorders.

There are two limitations of this study. First, the pedometer could not be worn during aqua exercise. Eighteen patients reported engagement in aqua exercise, such as swimming or aqua walking. Therefore, sport index may have had the lowest correlation coefficient, among BQ measurements. Second, the pedometer and BQ were different in terms of reference periods. The pedometer was used for only 5 to 7 days, whereas BQ referred to activities of no specified time component.

Previous studies have shown that mean BQ measures are 2.9 for work, 2.4 for sports, and 3.1 for leisure in women aged 20–32 years [10] and 2.7, 2.1, 2.6, and 7.4 (total) in older women (32% were 60 years or older) [32], suggesting that BQ can detect an age-related decline in multidimensions of physical activity. BQ measures of the study by Pols et al. were similar to those (2.8, 2.0, 2.6, and 7.5) of the present study despite a difference in age. People with hip disorders may be lower physical activity.

## Conclusion

In conclusion, BQ is a useful tool for assessing multiple domains of physical activity with acceptable reliability and validity in adult women who have hip disorders.

## Competing interests

The author(s) declare that they have no competing interests.

## Authors' contributions

RO carried out the validation and reliability study, participated in the sequence alignment and drafted the manuscript. SH, MK, YT conceived of the study, and participated in its design and coordination and helped to draft the manuscript. MY, TN, participated in acquisition of data at the outpatient's clinic and helped to interpret these data. All authors read and approved the final manuscript.

## Pre-publication history

The pre-publication history for this paper can be accessed here:


